# Correlating milk cytokines and somatic cell counts to intramammary infections in dairy sheep

**DOI:** 10.3389/fvets.2025.1711844

**Published:** 2026-01-05

**Authors:** Giulia Franzoni, Susanna Zinellu, Vittoria D’Ascenzo, Emanuela Giaconi, Giovanni Vito Denti, Marina Adele Lucia Manai, Angelo Fiori, Silvia Dei Giudici, Riccardo Bazzardi, Sara Casu, Antonello Carta, Ilaria Fadda, Ciriaco Ligios, Simone Dore

**Affiliations:** 1 Department of Animal Health, Istituto Zooprofilattico Sperimentale della Sardegna, Sassari, Italy; 2 National Reference Center for Sheep and Goat Mastitis, Istituto Zooprofilattico Sperimentale della Sardegna, Sassari, Italy; 3 Research Unit Genetics and Biotechnology, Agris Sardegna, Sassari, Italy

**Keywords:** biomarkers, chemokines, dairy sheep, IFN-*γ*, interleukins, mastitis, somatic cell counts

## Abstract

Mastitis represents an important health problem in dairy sheep and somatic cell count (SCC) is frequently used as indicator of subclinical intra-mammary infection. Cytokines might represent another reliable and sensitive tool for defining the inflammatory status in relation with the SCC, thus, the levels of 12 key immune cytokines were monitored in ovine milk samples with different SCC values. First, samples were divided into five SCC-groups: group 1 (0–300 cell/mL*1000, *n* = 16), group 2 (300–500 cell/mL*1000, *n* = 16), group 3 (500–1,000 cell/mL*1000, *n* = 16), group 4 (1000–2000 cell/mL*1000, *n* = 15), group 5 (> 2000 cell/mL*1000, *n* = 16). Samples belonging to group 5 presented the highest values of IL-1α, IL-*β*, IL-6, MIP-1α, MIP-1β, IFN-*γ*, IL-17, IL-10. Samples belonging to group 1 presented IL-4 levels higher than to those belonging to groups 2–3-4, and lower IL-1α and MIP-1α values compared to groups 3–4. Next, cluster analysis was performed. Three clusters were defined: cluster 1 (samples with low SCC values negative to bacterial culture), cluster 2 (samples positive to culture isolation with intermediate SCC values), cluster 3 (samples positive to culture isolation and with high SCC levels). Samples in cluster 1 were characterised by low levels of IFN-*γ*, IL-1α, IL-1β, IL-6, MIP-1α, whereas samples in cluster 3 presented the highest values of these five cytokines. Samples in cluster 2 presented higher levels of IL-1α and MIP-1α compared to cluster 1, as well as lower levels of IL-4. MIP-1α and IL-1α showed the strongest correlation with SCC values. Overall, our data revealed that intra-mammary infection in dairy sheep was correlated with high levels of pro-inflammatory cytokines in milk samples, which reflect the presence of an inflammatory reaction and confirm the utility of SCC determination in the diagnosis of subclinical mastitis.

## 1 Introduction

Mastitis due to bacterial intra-mammary infection is one of the most significant health problems in dairy sheep farming, which results in reduction of milk yield and quality, with subsequent relevant economic losses (1, 2). Therefore, proper diagnosis is a critical aspect of preventing mastitis in dairy sheep and its economic consequences (1–3).

Mastitis can be clinical or subclinical. The clinical form is characterized by visible abnormalities in milk (such as presence of blood, pus, color change) or alteration of the mammary gland, assessed by examination of the udder (1). On the contrary, identification of subclinical mastitis is more challenging. The diagnosis of subclinical mastitis is mainly performed on the milk through the Somatic Cell Count (SCC) determination, through flow cytometry (4), or indirectly by the California Mastitis Test (CMT) (5). During subclinical mastitis, there is indeed an inflammatory reaction characterized by influx of leukocytes in the udder, with subsequent increase of somatic cell number (both leukocytes and epithelial cells) in the milk, but without visual alterations in gland tissue or milk secretion (4). However, SCC present some disadvantages: this parameter could be influenced by several non-infectious factors, including age, breed, stage of lactation, milking interval, time of sampling, sampling procedures, stress and trauma, management factors, and seasonal and storage procedures (6).

Diagnostic performance can be increased by combination of somatic cell count and bacterial culture (1, 3). Bacteriological culture of milk is indeed a specific test for the diagnosis of contagious mastitis; it also allows the identification of the cause of inflammation. Nevertheless, its applicability as a mastitis screening tool is limited due to the cost/time required for its realization (3).

Other molecules might be used as mastitis biomarkers and improve diagnostic performance of mastitis in dairy sheep. Several antibacterial and immune defense proteins, including haptoglobin, serum, amyloid A, lactoferrin, and cathelicidin, increase significantly in milk upon infection and can potentially serve as mastitis biomarkers (7–10). For instance, a recent study showed that cathelicidin can be a useful milk biomarker of mastitis in sheep, presenting higher specificity compared to SCC (11).

Cytokines are pivotal elements of immune responses, and their evaluation provides insights into physiological and pathological processes, aiding diagnosis and treatment. These molecules have been widely studied as biomarkers for many diseases, considering that their quantification in biological fluids, such as milk, is characterised by lack of invasiveness and relative low cost (12).

Previous studies in cattle reported that pro-inflammatory cytokines and chemokines are produced and released at the early stages of infection of the mammary gland, to eliminate the invading pathogen (13–17). Anti-inflammatory cytokines are also secreted, to suppress inflammation and prevent the development of an exacerbated immune response (12, 13). Several studies have been conducted in cattle to evaluate whether these molecules could provide a reliable and sensitive diagnostic tool in mastitis (13–17), while knowledge about their role in sheep mastitis remains scarce (10).

In this study, levels of 12 key immune cytokines in sheep milk were measured alongside SCC, with the aim of providing preliminary insights into their potential role as biomarkers for mastitis in dairy sheep.

## 2 Materials and methods

### 2.1 Samples

Seventy-nine half-udder milk samples were included in the study. These samples were collected from February to June 2024 in a flock of Sarda ewes raised on an experimental farm in southern Sardinia, an area characterized by a semi-arid Mediterranean climate with marked seasonal and annual variation in rainfall and temperature. Management follows the island’s traditional farming system, relying on grazing natural or cultivated pastures, supplemented with hay, silage, and concentrates. Estrus are synchronized and most adult ewes lamb in autumn, and indeed the animals under study lambed in November 2023. After lamb separation, about 1 month postpartum, ewes are machine-milked twice daily until early summer, when they are progressively and almost simultaneously dried off. Information regarding month of milk sampling and temperature humidity index (THI) are reported in Table 1.

**Table 1:** Intra-mammary infection and somatic cell count levels in samples under study.

Month	Sample number	THI (°C)	Bacterial isolation	Cultural codification	SCC (10^3^/mL)	SCC Group
June	3	25.4	Negative	Negative	203	1
June	4	25.4	Negative	Negative	126	1
June	53	25.4	Negative	Negative	62	1
June	54	25.4	Negative	Negative	32	1
June	80	25.4	Negative	Negative	68	1
June	81	25.4	Negative	Negative	33	1
June	82	25.4	Negative	Negative	36	1
June	93	25.4	Negative	Negative	146	1
June	123	25.4	Negative	Negative	42	1
June	128	25.4	Negative	Negative	42	1
June	147	25.4	Negative	Negative	14	1
June	154	25.4	Negative	Negative	32	1
June	165	25.4	Negative	Negative	62	1
June	171	25.4	Negative	Negative	31	1
June	179	25.4	Negative	Negative	59	1
June	188	25.4	Negative	Negative	74	1
April	162	19.7	Corynebacterium spp.	Environmental	383	2
February	85	18.9	Staphylococcus petrasii	NAS	350	2
February	123	18.9	*Staphylococcus chromogenes*	NAS	435	2
June	92	25.4	Staphylococcus petrasii	NAS	323	2
June	101	25.4	*Corynebacterium mastitidis*	Environmental	303	2
June	107	25.4	Staphylococcus croceilyticus	NAS	476	2
June	156	25.4	Negative	Negative	430	2
June	172	25.4	Staphylococcus petrasii; *Staphylococcus auricularis*	NAS	356	2
May	14	20.1	*Enterococcus faecalis*	Environmental	464	2
May	42	20.1	*Enterococcus hirae*	Environmental	486	2
May	43	20.1	Staphylococcus petrasii	NAS	414	2
May	79	20.1	Staphylococcus petrasii	NAS	494	2
May	177	20.1	*Escherichia coli*	Environmental	337	2
May	178	20.1	*Escherichia coli*	Environmental	451	2
March	70	12.3	Negative	Negative	330	2
March	170	12.3	Staphyolococcuspetrasii	NAS	475	2
April	87	19.7	*Staphylococcus chromogenes*	NAS	723	3
February	84	18.9	Aerococcusviridians	Environmental	858	3
February	130	18.9	*Staphylococcus chromogenes*	NAS	517	3
June	90	25.4	Staphylococcus croceilyticus	NAS	749	3
June	106	25.4	*Enterococcus faecalis*; *Staphylococcus microti*	NAS	602	3
June	164	25.4	Staphylococcus petrasii	NAS	637	3
June	189	25.4	Sphingomonaspaucimobilis	Environmental	990	3
May	3	20.1	Staphylococcus petrasii	NAS	533	3
May	8	20.1	Staphylococcus borealis; *Staphylococcus lentus*; Staphylococcus petrasii	NAS	663	3
May	19	20.1	Staphylococcus borealis	NAS	728	3
May	21	20.1	Staphylococcus petrasii	NAS	894	3
May	136	20.1	*Enterococcus faecalis*	Environmental	867	3
May	182	20.1	Staphylococcus borealis	NAS	640	3
March	7	12.3	Pantoeaagglomerans, *Staphylococcus equorum*, Staphylococcus borealis	NAS	594	3
March	17	12.3	Staphylococcus petrasii	NAS	809	3
March	77	12.3	*Staphylococcus equorum*	NAS	580	3
April	1	19.7	*Enterococcus faecalis*	Environmental	1,202	4
April	18	19.7	*Staphylococcus microti*	NAS	1,713	4
April	21	19.7	*Enterococcus faecalis*	Environmental	1,542	4
April	57	19.7	*Staphylococcus chromogenes*	NAS	1,016	4
April	75	19.7	*Staphylococcus equorum*	NAS	1,037	4
June	12	25.4	*Staphylococcus simulans*	NAS	1,068	4
June	36	25.4	*Staphylococcus chromogenes*	NAS	1,925	4
June	39	25.4	*Staphylococcus chromogenes*	NAS	1,083	4
June	135	25.4	Staphylococcus borealis	NAS	1,040	4
June	155	25.4	Staphylococcus borealis	NAS	1,376	4
May	13	20.1	Staphylococcus petrasii	NAS	1,731	4
May	181	20.1	*Staphylococcus chromogenes*	NAS	1,872	4
March	50	12.3	*Staphylococcus chromogenes*	NAS	1,202	4
March	69	12.3	Sraphyloccousmicroti	NAS	1,414	4
March	85	12.3	*Streptococcus gallolyticus*	NAS	1,060	4
April	11	19.7	*Staphylococcus chromogenes*	NAS	4,371	5
April	124	19.7	*Staphylococcus gallinarum*; *Staphylococcus chromogenes*	NAS	25,075	5
April	143	19.7	*Staphylococcus gallinarum*	NAS	16,588	5
April	153	19.7	*Staphylococcus chromogenes*	NAS	2,403	5
February	71	18.9	*Staphylococcus xylosus*	NAS	12,919	5
June	29	25.4	*Staphylococcus chromogenes*	NAS	4,962	5
June	73	25.4	*Streptococcus gallolyticus*	NAS	10,568	5
June	119	25.4	Staphylococcus borealis	NAS	6,154	5
June	134	25.4	*Staphylococcus simulans*	NAS	6,917	5
June	142	25.4	Staphylococcus borealis	NAS	2,204	5
June	159	25.4	*Staphylococcus simulans*	NAS	3,664	5
June	160	25.4	Staphylococcus petrasii	NAS	2,411	5
May	60	20.1	Staphylococcus petrasii	NAS	7,551	5
May	83	20.1	Aerococcusviridians	Environmental	2,227	5
May	129	20.1	*Streptococcus gallolyticus*	NAS	22,509	5
May	161	20.1	*Staphylococcus simulans*	NAS	3,480	5

Milk samples under study were investigated for their SCC levels and presence of bacteria. Subsequently, milk samples were divided into five groups based on SCC values: group 1 (<300,000 SCC/mL, *n* = 16), group 2 (300,000–500,000 SCC/mL, *n* = 16), group 3 (500,000-1,000,000 SCC/mL, *n* = 16), group 4 (1,000,000-2,000,000 SCC/mL, *n* = 15), group 5 (> 2,000,000 SCC/mL, *n* = 16).

THI, temperature humidity index; SCC, somatic cell count.

Milk samples collection was performed in the context of routine voluntary herd screening programs for mammary gland health improvement by the farm veterinarians according to the standards set by the National Mastitis Council (18). In detail, before milk sampling, teats were carefully cleaned and disinfected using disposable towels embedded with chlorhexidine; foremilk was stripped and eliminated in a separate bucket to avoid the potential environmental spread of bacteria, and about 40 mL of milk was collected aseptically from each half-udder into sterile vials.

Samples were sent refrigerated to the diagnostic laboratories (Istituto Zooprofilattico Sperimentale della Sardegna) where somatic cell count (See 2.2) and culture isolation (see 2.3) were performed within 24 h. For cytokines determination, milk samples were stored at −80 °C until analyzed (See 2.4).

### 2.2 Somatic cell count and study design

The SCC was determined with an automated counter (Fossomatic FC; Foss Electric, HillerØd, Denmark) (UNI EN ISO 13366-2:2007) (19). Milk samples were divided into five groups based on SCC values: group 1 (<300,000 SCC/mL, *n* = 16), group 2 (300,000–500,000 SCC/mL, *n* = 16), group 3 (500,000–1,000,000 SCC/mL, *n* = 16), group 4 (1,000,000–2,000,000 SCC/mL, *n* = 15), group 5 (> 2,000,000 SCC/mL, *n* = 16).

### 2.3 Culture isolation

Milk samples were cultured following the standard procedures provided by the Istituto Zooprofilattico Sperimentale della Sardegna, as previously described (9, 20) and the National Mastitis Council guidelines (18). In detail, 10 μL of milk were seeded in 5% sheep blood agar, incubated at 37 °C, and examined after 24–48 h for colony growth. In case of bacteria growth, colonies were re-isolated in blood agar. Identification was finally performed by mass spectrometry technique with MALDI Biotyper® Sirius One System (Bruker) adopting the MBT Compass Library 2023 and MBT IVD Library 2023.

### 2.4 Cytokines release

Levels of 12 key immune cytokines (IL-1α, IL-1β, IL-4, IL-6, IL-10, IL-17, TNF, MIP-1α, MIP-1β, IL-36Ra, VEGF-A) were measured using Sheep Cytokine/Chemokine Magnetic Bead Panel Multiplex assay (Merck Millipore, Darmstadt, Germany) and a Bioplex MAGPIX Multiplex Reader (Bio-Rad, Hercules, CA, USA), following manufacturer instructions (21). All samples were tested in duplicate (two technical replicates).

### 2.5 Statistical analysis

The Shapiro–Wilk test was used to test the normal distribution of each independent variable. Data were then graphically and statistically analyzed with GraphPad Prism 10.01 (GraphPad Software Inc., La Jolla, CA, USA) and STATA/SE 19.0 (StataCorp LLC: College Station, TX). Median and inter-quartile range (IQR) were used to provide descriptive statistics. Differences between groups were evaluated using the non-parametric Kruskal–Wallis test with the Bonferroni’s correction.

A post-clustering hierarchical analysis was performed to visually assess the distribution of the clusters; the number of clusters was defined based on the 1 Gower dissimilarity value, which represents the distance measure aimed to calculate the distance between mixed (i.e., categorical and numerical) variables. Descriptive and inferential analyses were performed with the same test to detect the statistical differences between clusters.

Finally, a linear regression analysis was carried out using STATA/SE 19.0 to evaluate association between SCC values (in logarithmic scale) and selected cytokines; GraphPad Prism 10.01 was used to graphically represent the data. For all statistical analysis, the cutoff of *p*-value < 0.05 was considered statistically significant.

## 3 Results

### 3.1 Relationship of somatic cell counts and intramammary infection

All 16 samples belonging to group 1 (<300,000 SCC/mL) were negative to culture isolation, whereas 14 out of 16 milk samples belonging to group 2 (300,000–500,000 SCC/mL) were positive to culture isolation, and all the samples belonging to the other groups (3-4-5) were positive to culture isolation. Bacteria identified were either *Non*-*aureus* staphylococci (NAS) or environmental causative agents of mastitis (i.e.*, Corynebacterium* spp.*, Enterococcus* spp., *Aerococcus* spp.*, Sphingomonas* spp.*, Escherichia coli*), as presented in Table 1.

### 3.2 Relationship of SCC and cytokines milk levels

**Figure 1:**
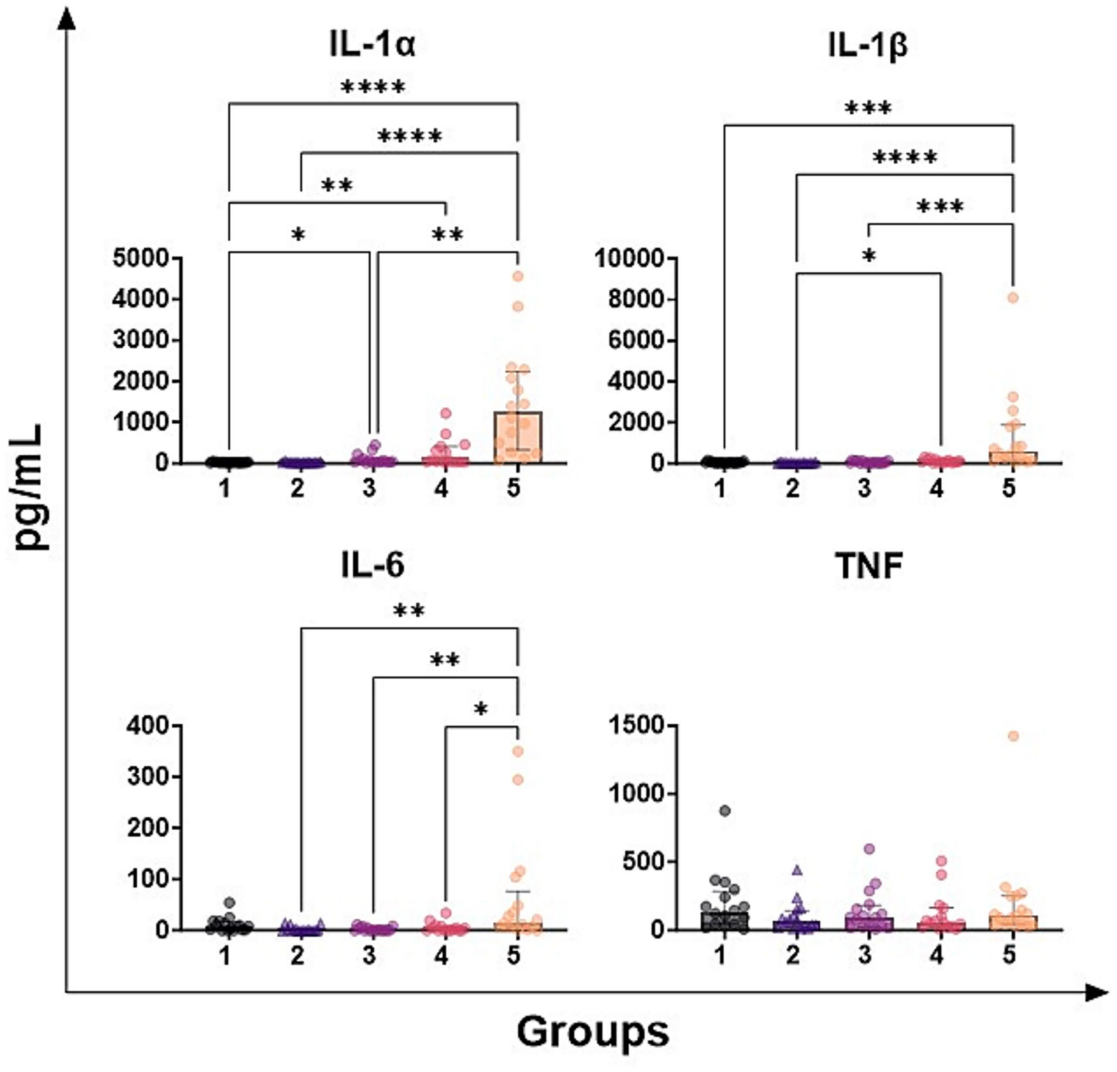
Levels of chemokines in milk samples with diverse somatic cell count values. Seventy-nine ovine half-udder milk samples were included in this study. Somatic cell count (SCC) were determined using an automated counter and then milk samples were divided into five groups: group 1 (< 300,000 SCC/mL, *n* = 16), group 2 (300,000–500,000 SCC/ml, *n* = 16), group 3 (500,000-1,000,000 SCC/mL, *n* = 16), group 4 (1,000,000-2,000,000 SCC/mL, *n* = 16), group 5 (> 2,000,000 SCC/mL, *n* = 16). Levels of IL-1α, IL-1β, IL-6, and TNF were quantified through multiplex ELISA. Differences between groups were evaluated using a Kruskal–Wallis test; * *p* < 0.05, ** *p* < 0.01, *** *p* < 0.001, **** *p* < 0.0001.

Levels of four key pro-inflammatory cytokines (IL-1α, IL-1β, IL-6, TNF) in the milk samples under study were quantified by ELISA and our data revealed differences between samples belonging to diverse groups. As presented in Figure 1, we observed that milk samples with the highest SCC levels (group 5) presented higher IL-1α and IL-*β* values compared to those with lower SCC (groups 1–2-3). In a similar way, milk samples belonging to group 5 presented statistically higher values of IL-6 compared to those with lower SCC, also between group 4 and group 5 (Figure 1). On the contrary, no statistical differences between groups were detected for TNF (Figure 1). Regarding IL-1α, differences were also observed between samples belonging to group 1 (negative to culture isolation, < 300,000 SCC/mL) and to those with intermediate SCC values (group 3; 500,000-1,000,000 SCC/mL) (Figure 1).

**Figure 2:**
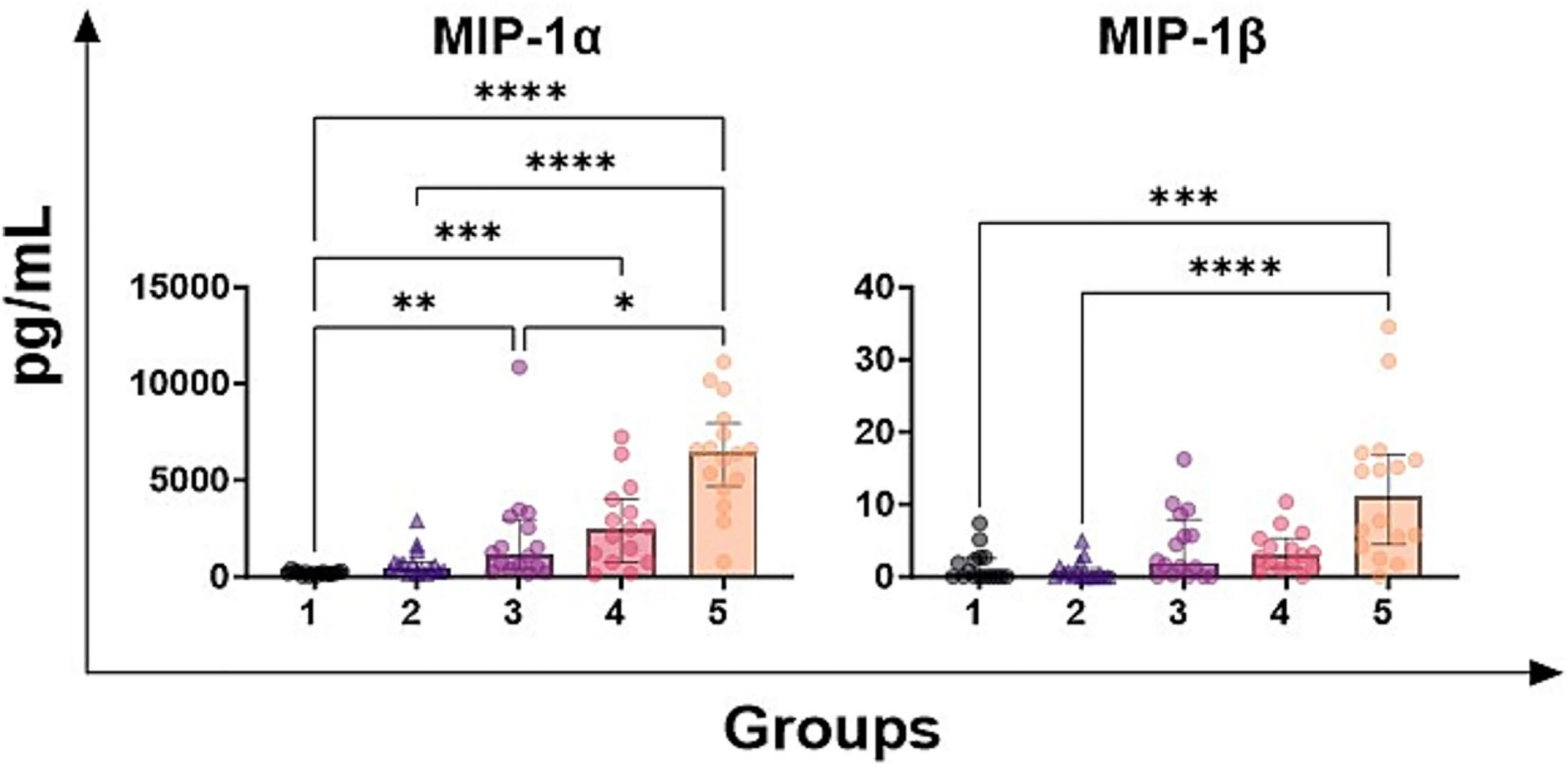
Levels of chemokines in milk samples with diverse somatic cell count values. Seventy-nine ovine half-udder milk samples were included in this study. Somatic cell count (SCC) were determined using an automated counter and then milk samples were divided into five groups: group 1 (<300,000 SCC/mL, *n* = 16), group 2 (300,000–500,000 SCC/ml, *n* = 16), group 3 (500,000-1,000,000 SCC/mL, *n* = 16), group 4 (1,000,000-2,000,000 SCC/mL, *n* = 16), group 5 (> 2,000,000 SCC/mL, *n* = 16). Levels of MIP-1α and MIP-1β were quantified through multiplex ELISA. Differences between groups were evaluated using a Kruskal–Wallis test; * *p* < 0.05, ** *p* < 0.01, *** *p* < 0.001, **** *p* < 0.0001.

Levels of two key pro-inflammatory chemokines were next investigated: MIP-1α and MIP-1β. We observed that samples with high SCC values (group 5) presented higher milk levels of both MIP-1α and MIP-1β compared to those with lower SCC belonging (groups 1–2) (Figure 2). Regarding MIP-1α, but not MIP-1β, differences were also observed between samples belonging to group 1 and groups 3 and 4 (Figure 2).

**Figure 3:**
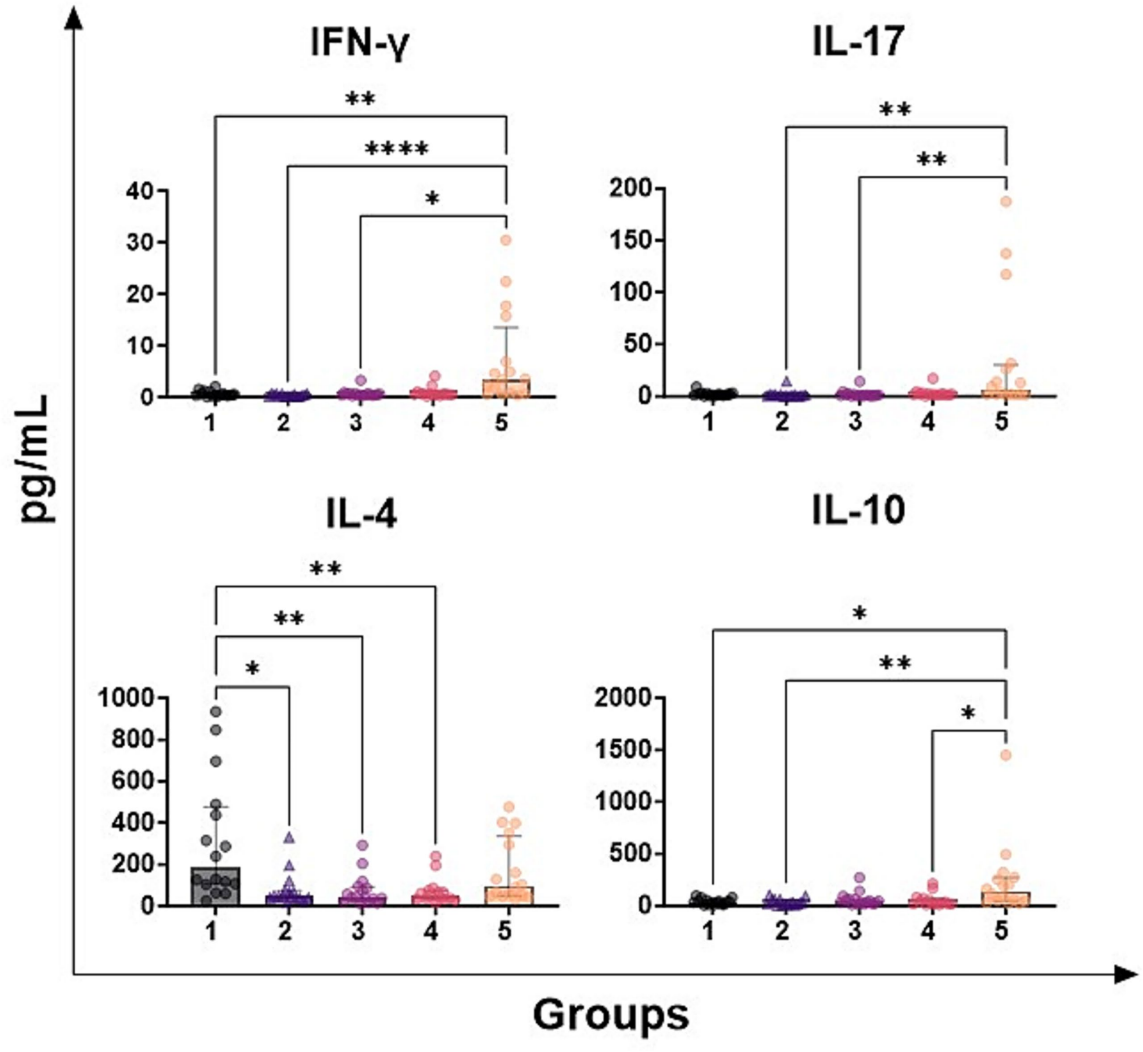
Levels of T-cell cytokines in milk samples with diverse somatic cell count values. Seventy-nine ovine half-udder milk samples were included in this study. Somatic cell count (SCC) were determined using an automated counter and then milk samples were divided into five groups: group 1 (<300,000 SCC/mL, *n* = 16), group 2 (300,000–500,000 SCC/ml, *n* = 16), group 3 (500,000-1,000,000 SCC/mL, *n* = 16), group 4 (1,000,000-2,000,000 SCC/mL, *n* = 16), group 5 (> 2,000,000 SCC/mL, *n* = 16). Levels of IFN-*γ*, IL-4, IL-10, and IL-17 were quantified through multiplex ELISA. Differences between groups were evaluated using a Kruskal–Wallis test; * *p* < 0.05, ** *p* < 0.01, **** *p* < 0.0001.

Subsequently, levels of four T-cell cytokines in milk samples were quantified: IFN-*γ* (hallmark of Th1 response), IL-4 (hallmark of Th2 response), IL-17A (hallmark of Th17 response), IL-10 (immunosuppressive cytokines related to regulatory T cells). We observed that samples with high SCC values (group 5) presented higher milk levels of IFN-γ, IL-17A, IL-10, but not IL-4, compared to those with low SCC (Figure 3). For IL-10, statistically significant differences were observed also between group 4 and 5 (Figure 3). On the contrary, samples in group 1 presented higher levels of IL-4 compared to milk samples belonging to group 2–3-4 but not group 5 (Figure 3).

**Figure 4:**
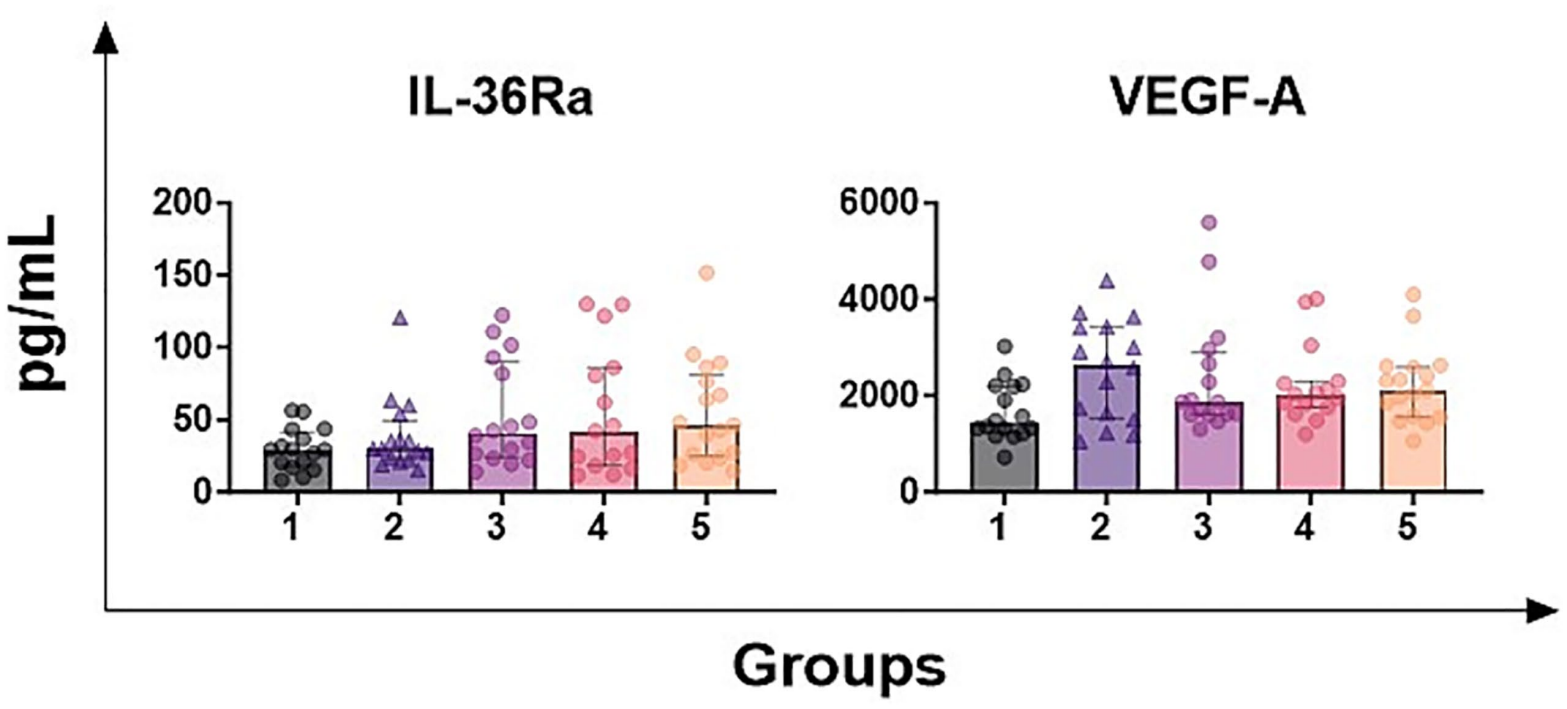
Levels of IL-36Ra and VEGF in milk samples with diverse somatic cell count values. Seventy-nine ovine half-udder milk samples were included in this study. Somatic cell count (SCC) were determined using an automated counter and then milk samples were divided into five groups: group 1 (<300,000 SCC/mL, *n* = 16), group 2 (300,000–500,000 SCC/ml, *n* = 16), group 3 (500,000-1,000,000 SCC/mL, *n* = 16), group 4 (1,000,000-2,000,000 SCC/mL, *n* = 16), group 5 (> 2,000,000 SCC/mL, *n* = 16). Levels of IL-36Ra and VEGF-A were quantified through multiplex ELISA. Differences between groups were evaluated using a Kruskal–Wallis test; * *p* < 0.05, ** *p* < 0.01, *** *p* < 0.001, **** *p* < 0.0001.

Next, we monitored the levels of the receptor antagonist IL-36Ra, which is secreted to prevent the development of an exacerbated inflammatory response to stressors (22), and the growth factor VEGF-A, which promote angiogenesis and increase vascular permeability (23). As presented in Figure 4, for these two cytokines no statistically statistical differences between groups were detected.

### 3.3 Cluster analysis

**Figure 5:**
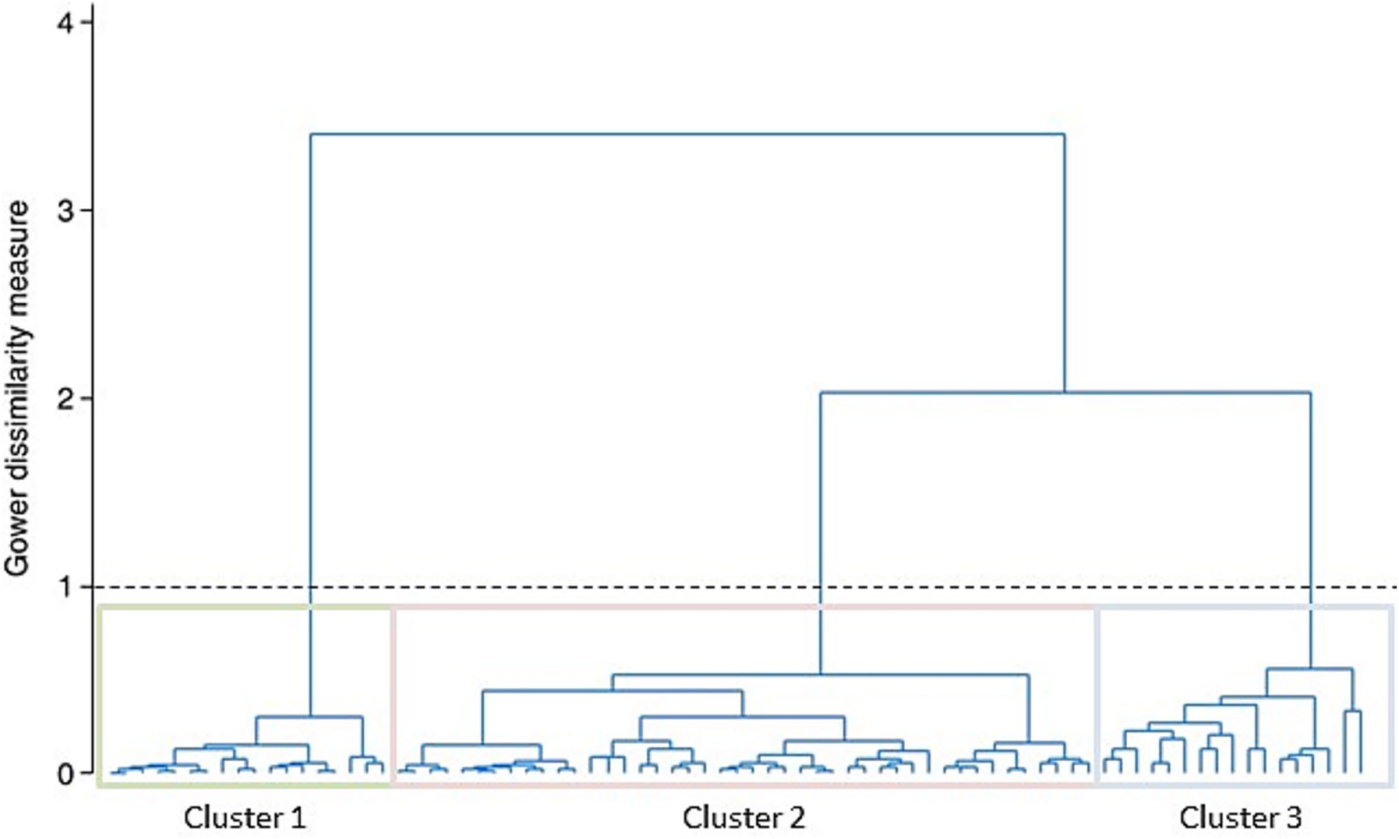
Dendrograms of the cluster analysis. Cluster analysis was performed using several variables: bacteriological culture data, SCC-groups, and levels of the 12 key immune cytokines. The number of clusters was defined based on the 1 Gower dissimilarity value.

Cluster analysis was then carried out using bacteriological culture data (negative/positive; infection with NAS or environmental agents of mastitis), SCC groups, and levels of the 12 key immune cytokines. Three clusters were defined based on the 1 Gower dissimilarity value, as presented in Figure 5.

Cluster 1 included 22.78% (*n* = 18) of the samples and all of them were negative to culture isolation (Table 2). In comparison with the other two clusters, these samples presented low levels of SCC. In details, 88.89% (*n* = 16) of samples belonging to this cluster presented SSC values lower than 300,000 cells/mL and 11.11% (*n* = 2) belonged to the SSC-Group 2 (300,000–500,000 cells/mL) (Table 3). Based on these characteristics, Cluster 1 was labeled as “healthy.”

**Table 2:** Intramammary infection in the three clusters.

Bacterial culture	Cluster		
	Cluster 1	Cluster 2	Cluster 3
Negative	18 (100%)	0 (0%)	0 (0%)
Positive / environmental	0 (0%)	12 (27.27%)	32 (72.73%)
Positive / NAS	0 (0%)	0 (0%)	17 (34.69%)

For each cluster, results of bacterial culture tests are presented.

**Table 3:** SCC levels in the samples belonging to the three clusters.

SSC group	Cluster		
	Cluster 1 (Healthy)	Cluster 2 (Intermediate SCC)	Cluster 3 (High SCC)
Group 1 (< 300,000 cells/mL)	16 (88.89%)	0 (0%)	0 (0%)
Group 2 (300,000–500,000 cells/mL)	2 (11.11%)	14 (31.82%)	0 (0%)
Group 3 (500,000 – 1,000,000 cells/mL)	0 (0%)	16 (36.36%)	0 (0%)
Group 4 (1,000,000 – 2,000,000 cells/mL)	0 (0%)	13 (29.55%)	2 (11.76%)
Group 5 (> 2,000,000 cells/mL)	0 (0%)	1 (2.27%)	15 (88.24%)

For each cluster, samples belonging to different SCC-groups are presented.

Cluster 2 included 55.70% (*n* = 44) of the samples and all of them were positive to culture isolation. In details, 27.27% (*n* = 12) of these were samples positive to environmental causative agents of mastitis and 72.23% (*n* = 32) were positive to NAS (Table 2). In comparison with the other two clusters, these samples presented intermediate levels of SCC. In details, 31.82% (*n* = 14) of these samples belonged to the SSC-Group 2 (300,000–500,000 cells/mL), 36.36% (*n* = 16) to the SSC-Group 3 (500,000-1,000,000 cells/mL), 29.55% (*n* = 16) to the SSC-Group 4 (1,000,000–2,000,000 cells/mL), and only 2.27% (*n* = 1) to SSC-Group 5 (>2,000,000 cells/mL) (Table 3). Based on these characteristics, Cluster 2 was named “intermediate SCC.”

Cluster 3 included 21.52% (*n* = 17) of the samples and all of them were positive to culture isolation. All the samples belonging to this cluster were samples positive to environmental causative agents of mastitis (Table 2). In comparison with the other two clusters, these samples presented higher levels of SCC. In details, 88.24% (*n* = 15) of these samples belonged to the SSC-Group 5 (> 2 000,000 cells/mL) and 11.76% (*n* = 2) to the SSC-Group 4 (1,000,000–2,000,000 cells/mL). Based on these characteristics, Cluster 3 was named “high SCC.”

Inferential analyses between clusters were performed to detect statistical differences in terms of key immune cytokines. Our analysis revealed significant differences between these clusters for six cytokines: IFN-*γ*, IL-1α, IL-1β, IL-4, IL-6, MIP-1α (Table 4). No significant differences were instead observed for the other six cytokines analysed: IL-10, IL-17, TNF, MIP-1β, IL-36Ra, and VEGF-A (Table 4).

**Table 4:** Cytokines profile in the three clusters.

Variable	Cluster			*p*-value
	Cluster 1 (Healthy) Median (IQR)	Cluster 2 (Intermediate SCC) Median (IQR)	Cluster 3 (High SCC) Median (IQR)	
IFN-γ	0.436 (0.275–0.682)	0.48 (0.28–0.63)	3.40 (0.80–6.88)	0.0001
IL-1α	15.34 (10.93–29.32)	32.72 (20.23–67.13)	1215.27 (713.49–2072.83)	0.0001
IL-1β	39.90 (22.23–59.76)	43.95 (24.98–92.49)	497.92 (185.36–1807.00)	0.0001
IL-4	127.14 (67.12–438.10)	47.66 (36.53–80.95)	89.12 (46.73–294.97)	0.0003
IL-6	6.32 (0.16–14.08)	3.96 (0.61–5.44)	12.42 (6.99–35.63)	0.0002
IL-10	38.95 (13.22–62.87)	28.21 (15.22–55.72)	161.15 (56.02–253.88)	> 0.05
IL-17	1.31 (0.60–2.23)	1.17 (0.50–1.74)	3.78 (1.73–26.06)	>0.05
MIP-1α	204.42 (113.3–239.30)	908.06 (354.33–2534.75)	6552.61 (5345.62–7379.04)	0.0001
IL-36RA	29.5 (20.41–43.28)	34.78 (23.00–62.79)	48.14 (27.46–86.36)	>0.05
MIP-1β	0 (0–2.30)	1.52 (0.12–4.30)	10.34 (5.45–16.13)	>0.05
TNF	149.5 (47.47–242.98)	59.01 (23.98–113.14)	110.79 (48.22–251.19)	>0.05
VEGF-A	1420.85 (1213.41–2188.73)	2189.93 (1676.88–3123.47)	2031.55 (1545.46–2331.53)	>0.05

For each cluster, median and interquartile range values for each cytokine are presented. Descriptive and inferential analyses between clusters were performed to detect statistical difference.

IQR, inter quartile range.

In details, samples in cluster 1 were characterized by low levels of IFN-*γ*, IL-1α, IL-1β, IL-6, MIP-1α, whereas samples in cluster 3 presented the highest levels of these five cytokines. Samples in cluster 2 presented higher levels of IL-1α and MIP-1α compared to cluster 1. In addition, samples in cluster 2 showed lower levels of IL-4 compared to the other two clusters (Table 4).

### 3.4 Relationship between somatic cell count and selected cytokines

**Figure 6:**
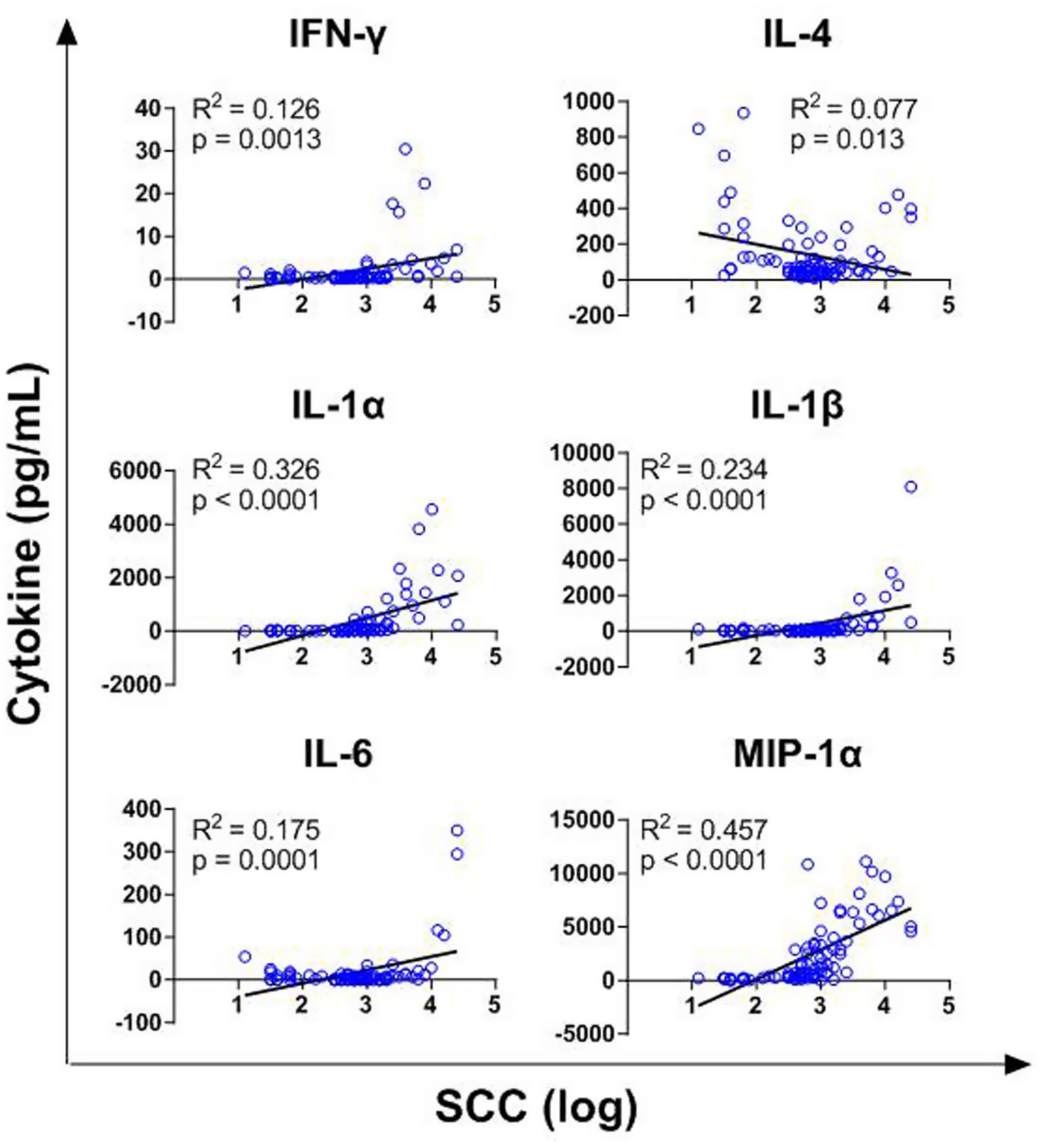
Linear regression analysis of selected cytokines with somatic cell count values. A linear regression analysis was performed considering SCC values (in logarithmic scale) as independent variable, and IFN-γ, IL-1α, IL-1β, IL-4, IL-6, MIP-1α as dependent variables. For each cytokines, R^2^ and *p* values are presented.

Finally, the association between SCC values and the six cytokines presenting differences between clusters (IFN-γ, IL-1α, IL-1β, IL-4, IL-6, MIP-1α) was evaluated. A linear regression analysis was performed considering SCC values as independent variable, and IFN-γ, IL-1α, IL-1β, IL-4, IL-6, MIP-1α as dependent variables. As presented in Figure 6, all these cytokines were significantly associated with SCC values, with only IL-4 displaying a negative regression coefficient. MIP-1α and IL-1α showed the highest coefficients of determination (R^2^ = 0.457 and R^2^ = 0.326, respectively), whereas IL-4 presented the lowest (R^2^ = 0.077) (Figure 6). Regarding IL-4, the relatively low R^2^ was due to high cytokine values being observed both in samples with low and extremely high SCC values (Figure 6).

## 4 Discussion

Mastitis is an inflammatory disease of the mammary gland characterised by recruitment of leukocytes and release of cytokines. Increased levels of somatic cells (leukocytes and epithelial cells) in milk samples are routinely used in the diagnosis of mastitis in both bovine and small ruminants (1). Nevertheless, there is no consensus on the SCC cut-off value for sheep milk able to discriminate between a healthy udder and those with subclinical mastitis (8). Rosati and collaborators suggested a SCC cut-off of 265,000 cells/mL (24) and similarly Albenzio and collaborators observed that SCC values > 300,000 cells/mL resulted in decreased milk production in sheep (6). Other researchers reported that the limit of 400,000 SCC/mL (25, 26) or even 500,000 SCC/mL (7, 27) would be most suitable to detect subclinical mastitis in sheep. In agreement, we observed that all milk samples with SCC < 300,000 cells/mL were negative for bacterial culture, while 2 out of 16 were included in the range 300,000–500,000 cells/mL.

As above stated, several factors can affect SCC values in sheep, such as age, parity, breed, management system, stress, physiological lactation stage, and season (6, 8). Therefore, it would be helpful to identify mastitis biomarkers less affected by non-infectious factors. Very few studies investigated the role of cytokines as potential diagnostic biomarkers for mastitis in small ruminants and in our study, we provided preliminary insights into the role of 12 key immune cytokines as biomarkers for mastitis in dairy sheep.

First, milk levels of four pro-inflammatory cytokines were analysed: IL-1α, IL-1β, IL-6, TNF. These cytokines are typically released early during infection to initiate inflammation. They trigger the release of release of pro-inflammatory chemokines, which effectively recruit leukocytes to the inflammatory site (22, 28). As expected, samples with the highest values of SCC presented also the highest levels of IL-1α, IL-1β, IL-6. These samples likely possess the highest values of leukocytes, recruited from the in the mammary gland in case of infection, which release high levels of these pro-inflammatory cytokines to boost inflammation (14). Similar results were indeed observed in cattle: infection with either Gram positive or Gram-negative bacteria resulted in early release of these pro-inflammatory cytokines (13–15). Among leukocytes, macrophages are likely the main source of IL-1β, whereas mammary epithelial cells can also be an important source of IL-6 (29). Surprisingly, we did not observed differences between SCC-groups in terms of TNF, whereas high levels of this cytokine were observed in bovine milk samples with high SCC values (15). Further studies on a larger set of samples should be performed to understand differences between these two species.

Chemokines are a group of cytokines primarily involved in recruiting immune cell recruitment to infected tissues. MIP-1α and MIP-1β are two pro-inflammatory chemokines mainly produced by monocytes/macrophages, whose main function is to promote recruitment of diverse cell types to the inflammatory sites (30). As expected, milk samples with the highest values of SCC presented also the highest levels of these two chemokines. The release of these chemokines during mastitis triggers influx of leukocytes from the bloodstream and thus increases SCC levels in milk samples (31).

IFN-γ is a type II IFN mainly released by NK and activated T cells; it is considered a hallmark of a Th1 response, which is associated with resistance to intracellular pathogens (32). On the contrary, IL-4 and IL-17 are regarded as hallmark of the Th2 and Th17 response, respectively (28, 33). Samples with the highest SCC values presented also elevated levels of IFN-γ, IL-17, IL-10.

In cattle, high IFN-γ values were observed in milk samples collected from udder with persistent infection (14). A Th1 response is generally activated at later stage of mammary infection, when innate immune defenses fail to promptly eliminate the invading pathogens (14). It is likely that IFN-γ levels in milk samples could be a hallmark of persistent infection in dairy sheep, although this hypothesis should be validated on a larger number of samples with the association of more detailed clinical and epidemiological investigations.

The highest IL-17 values were observed in milk samples belonging to group 5, characterised by high IFN-γ levels, suggesting that a Th17 response was often activated in parallel to a Th1 response. Nevertheless, no statistical differences between group 5 and 1 were observed, suggesting that IL-17 unlikely represent potential diagnostic biomarker for mastitis in dairy sheep.

IL-10 is a potent anti-inflammatory cytokine, released to reduce or terminate inflammation (28). We observed higher levels of IL-10 in samples with the highest levels of SCC (> 2,000,000 cells/mL) and this is like what observed in cattle: infection with either Gram positive or Gram negative bacteria resulted in release also of this anti-inflammatory cytokine (14). The highest IL-10 values were observed in samples characterised also by high levels of pro-inflammatory cytokines, suggesting that this cytokine is likely released mainly as a defence mechanism, to avoid the development of an exacerbated immune response. Nevertheless, no differences between group 5 and 3 were observed, suggesting that IL-10 unlikely represent potential diagnostic biomarker for mastitis in dairy sheep.

Interestingly, samples belonging to SSC-group 1 were characterised by IL-4 levels higher than samples belonging to groups 2–3-4. Previous studies reported that IL-4 promotes mammary gland development (34) and proliferation of mammary epithelial cells (35). Probably intra-mammary infection negatively affects the physiological production of IL-4 in the mammary gland, with subsequent drop of IL-4 levels in the milk. Nevertheless, no differences were observed between group 1 and 5. Several samples belonging to SCC-group 5 (SCC > 2,000,000 cells/mL) presented also the highest IFN-*γ* values, which might be a sign of persistent infection, and we think that elevated IL-4 levels in these samples reflect the activation of a Th2 response. Nevertheless, this hypothesis needs a deeper investigation, with future studies including the differential somatic cell count (DSCC) parameter. DSCC provide information on the proportion of different cell types, such as granulocytes, macrophages, lymphocytes, and epithelial cells (36, 37) and it can help to better understand the differences of IL-4 between groups and to identify the cellular source of this cytokine in the different groups. Subsequently, we investigated the levels of IL-36Ra and VEGF. IL-36Ra is the receptor antagonist released to avoid development of an exacerbated inflammatory response (22). VEGF is a growth factor with pro-angiogenic activity, which promotes endothelial cell survival and vascular permeability (23). No differences between SCC-groups were observed, suggesting that their levels are not influenced by inflammation of the mammary gland, thus they unlikely represent potential diagnostic biomarker for mastitis in dairy sheep.

To better understand whether patterns of cytokine were able to discriminate the samples under study, cluster analysis was performed. Three clusters were defined: cluster 1 (samples negative to bacterial culture and with low SCC values), cluster 2 (samples positive to culture isolation and with intermediate SCC levels), cluster 3 (samples positive to culture isolation and with high SCC levels). Samples in cluster 3 (‘high SCC values’) included only NAS-positive samples, whereas cluster 2 (‘intermediated SCC values’) included 44 milk samples, 12 of which positive at environmental causative agents of mastitis (*Corynebacterium* spp., *Enterococcus* spp.*, Aerococcus*spp., *Sphingomonas spp., Escherichia coli*). Inferential analyses revealed that six cytokines were able to discriminate these three clusters: IFN-*γ*, IL-1α, IL-1β, IL-4, IL-6, MIP-1α.

Samples in cluster 3 presented the highest values of IFN-γ, which is likely associated to persistent infection. Previous studies reported that Gram negative bacteria (e.g., *Escherichia coli*) quickly trigger strong inflammation of the mammary gland, which activate a protective immune response that often eradicates the pathogen. On the contrary, Gram-positive bacteria slowly elicit a much weaker inflammation and immune response, frequently resulting in chronic infections (37). In agreement, we observed that samples belonging to cluster 3 included only NAS-positive milk samples, and it is likely that the 17 samples in cluster 3 with high IFN-γ values were collected from udder with persistent infection. Nevertheless, the potential use of IFN-γ as biomarker of persistent infection should be validated on a larger number of samples, with the association of more detailed investigations.

Pro-inflammatory cytokines are released more intensely after infections with Gram negative bacteria compared to Gram positive bacteria (38), nevertheless samples in cluster 2 presented lower levels of IL-1α, IL-*β*, IL-6, MIP-1α compared to those in cluster 3. This discrepancy is likely since samples in our study were selected randomly, and not at a specific time post-infection.

Interestingly, samples in cluster 2 (‘intermediated SCC values’) presented higher levels of IL-1α and MIP-1α compared to healthy samples (cluster 1), and lower levels of IL-4. These preliminary data suggest that determination of IL-1α, MIP-1α, IL-4 in sheep milk samples could contribute to the diagnosis of subclinical mastitis.

MIP-1α and IL-1α were positively associated with SCC values and presented the highest R^2^ values among the tested cytokines. IL-4 presented a low R^2^, perhaps because this cytokine is released by diverse cell types in samples with low and high SCC values. Future studies should be focused on these cytokines, in association with the DSCC parameter, to associate their changes with the levels of epithelial cells and diverse immune cell populations.

In the future, our preliminary results should be validated on a larger number of samples, especially those with SCC between 300,000–1,000,000, alongside clinical and epidemiological investigations, to evaluate whether factors like age, parity, breed, lactation stage, and season can influence these cytokines. In addition, a detailed analysis of the kinetics of these cytokines over time in milk samples from sheep with subclinical mastitis should be carried out.

Overall, our data revealed that intra-mammary infection was correlated with high levels of pro-inflammatory cytokines in milk samples, which reflect the presence of an inflammatory reaction, characterized by influx of leukocytes in the udder and subsequent increase of somatic cell number in the milk. Our data confirm the utility of SCC determination in the diagnosis of subclinical mastitis, and its combination with selected cytokines might improve our understanding of immune-pathogenetic mechanisms beyond mastitis in dairy sheep.

## Data Availability

The raw data supporting the conclusions of this article will be made available by the authors, without undue reservation.
